# The location of the inferior angle of the scapula in relation to the spine in the upright position: a systematic review of the literature and meta-analysis

**DOI:** 10.1186/s12998-014-0050-7

**Published:** 2015-02-27

**Authors:** Robert Cooperstein, Michael Haneline, Morgan Young

**Affiliations:** Palmer West College of Chiropractic, 90 East Tasman Drive, San Jose, CA 94577 USA; University of Western States, 2900 NE 132nd Avenue, Portland, OR 97230 USA

## Abstract

Practitioners in several of the health care professions use anatomical landmarks to identify spinal levels, both in order to enhance diagnostic accuracy and to specifically target the site of intervention. Authoritative sources usually state the upright inferior scapular angle (IAS) aligns with the spinous process (SP) of T7, but some specify the T7-8 interspace or the T8 SP. The primary goals of this study were to systematically review the relevant literature; and conduct a meta-analysis of the pooled data from retrieved studies to increase their statistical power. Electronic searching retrieved primary studies relating the IAS to a spinal level, as determined by an imaging reference standard, using combinations of these search terms: scapula, location, landmark, spinous process, thoracic vertebrae, vertebral level, palpation, and spine. Only primary studies were included; review articles and reliability studies related to scapular position but lacking spinal correlations were excluded. Eight-hundred and eighty (880) articles of interest were identified, 43 abstracts were read, 22 full text articles were inspected, and 5 survived the final cut. Each article (with one exception) was rated for quality using the QUADAS instrument. Pooling data from 5 studies resulted in normal distribution in which the upright IAS on average aligns closely with the T8 SP, range T4-T11. Since on average the IAS most closely identifies the T8 SP in the upright position, it is very likely that health professionals, both manual therapists and others, who have been diagnosing and treating patients based on the IAS = T7 SP rule (the conventional wisdom), have not been as segmentally accurate as they may have supposed. They either addressed non-intended levels, or made numeration errors in their charting. There is evidence that using the IAS is less preferred than using the vertebra prominens, and may be less preferred than using the iliac crest for identifying spinal levels Manual therapists, acupuncturists, anesthesiologists, nurses, and surgeons should reconsider their procedures for identifying spinal sites in light of this modified information. Inaccurate landmark benchmark rules will add to patient variation and examiner errors in producing spine care targeting errors, and confound research on the importance of specificity in treating spinal levels.

**Electronic supplementary material:**

The online version of this article (doi:10.1186/s12998-014-0050-7) contains supplementary material, which is available to authorized users.

## Introduction

Practitioners in several of the health care professions use anatomical landmarks to identify spinal levels, both in order to enhance diagnostic accuracy and to specifically target the site of intervention. Manual therapists palpate spinal and pelvic structures to determine both their position and their movement capacities. Anesthesiologists require precise placement of thoracic epidural catheters to optimize postoperative analgesia and minimize adverse effects [1-6]. Anatomic landmarks are also used to locate acupuncture points [7]; surgeons may decide upon a location to begin their incision based at least in part on the location of the IAS ([8] p.18).

Manual therapists use static palpation to identify asymmetry of bilateral structures, such as the posterior superior iliac spines [9,10]; as well as to identify malposition of contiguous structures, such as that of a spinal motion segment. Motion palpation is used to identify quantitative limitation in the excursion of contiguous structures, or changes in the qualitative properties of an osseous structure that has its movement taken to endrange [11]. Manual therapists generally believe that the confluence of both static and motion findings of abnormality establishes criteria for clinical intervention [12]. Indices of interexaminer agreement were generally found to be low in systematic reviews of both motion palpation [13,14] and static palpation [13], although more recent studies in which examiners determined the “most fixated level” rather than rating specific levels as fixated or not have shown high reliability in both the thoracic [15] and cervical [16] spinal regions.

The various health professions that make use of spinal palpation deploy a litany of spinal and pelvic landmarks to target potential sites of care, as well as chart levels that have been identified or treated. Since these anatomical landmarks are thought to identify corresponding spinal levels, other spinal levels may be located by counting up or down. Some of the most commonly used landmarks used in this way are C7, usually regarded to be the vertebra prominens (but not always) [17]), with the longest cervical spinous process; L4, whose SP is generally thought level with the iliac crest [18]; and S2, thought level with the posterior superior iliac spines [19]. Investigators have reported frequent mistakes in numerating spinal levels [20,21]. Some of these no doubt result from examiner palpatory errors [13,22], while others may result from variations in patient anatomy [17-19,23]. However, errors may also result from anatomical benchmark rules that are inherently inaccurate [24].

In chiropractic education it is commonly taught that the inferior angle of the scapula (IAS) can be located using the rule “7 up, 6 down,” referring to the position of the IAS in relation to the thoracic SPs in the upright and prone positions, respectively [25-27]. Spot checking, the T7 SP = IAS benchmark can also be found in other professions: anesthesiology [4,28], physiatry [29], orthopedic medicine [30,31], kinesiology [32], acupuncture [7], and nursing [33]. Other sources state the IAS line up with the T7-8 interspace [34,35], T8 SP, [36,37], or with the T9 SP [38]. The primary goal of this study was to conduct a systematic review of primary studies that addressed the location of the IAS in relation to the upright spine. The secondary goal was to perform meta-analysis using the combined data to increase the level of confidence that could be attached to the individual study findings.

## Methods

Table 1 uses the STARLITE mnemonic [39] to summary our search strategy. The titles, abstracts, and (as required) full publications of retrieved citations were independently screened by two reviewers. Any possible discrepancies were resolved by consensus. To be included, a study had to provide data on the location of the IAS in relation to the spine in the upright position, as determined by an imaging reference standard. A study could report the location of the IAS in relation to an SP, an intervertebral space, or a vertebral body. The investigators excluded review articles, reliability studies related to scapular position but not correlated with spinal levels; and studies that involved fractures, dislocations or congenital abnormalities of the scapula (e.g., Klippel-Feil syndrome and Sprengel deformity). Cadeveric and intraoperative studies were also excluded. Once a relevant citation was found, the “related citations” (or equivalent, depending on the website or database) function was deployed to find additional articles. A secondary search was also conducted using the included articles’ references. Each of the included articles, except Haneline [40] (which was an anatomy study and did not include a palpation arm) was rated for quality using the QUADAS index [41,42]. Scoring differences were discussed and, where necessary, resolved by a third reviewer. Meta-analysis was conducted on the combined data from the included studies and analysis, and presented as a forest plot in Figure three (Open Meta-Analyst, available from http://www.cebm.brown.edu/open_meta). Due to differences in how the included studies were done, pooling the data in some cases required transforming their findings, which may have related the scapula to an intervertebral space or a vertebral body, to correspond with a spinous process (SP) level. These transformations are described in the results section.

**Table 1:** **STARLITE search strategy summary**

**Sampling strategy**	**Electronic databases searched for articles satisfying inclusion criteria**
Type of studies	Anatomical studies investigating the spinal level corresponding to the scapula
Approaches	“Related articles” function used following successful retrieval. Secondary search to reach point of data saturation. Google searching.
Range of years	No restrictions.
Limits	Only English-language articles were included.
Inclusions/Exclusions	Included only primary studies where the spinal level corresponding to the scapula was identified through comparison with an imaging reference standard. Reliability studies and reviews of the literature were excluded. Intraoperative and cadaveric studies were excluded.
Terms used	Combinations of Non-MesH terms (Spinous Process, Thoracic Vertebrae, Vertebral Level, Validity) and MeSH terms (Scapula, Spine, Palpation, Diagnostic Techniques and Procedures, Diagnostic Imaging, Physical Examination, Anatomic Landmarks, Thorax, Reproducibility of Results).
Electronic sources	PubMed, MANTIS, ICL, CINAHL, AMED, Osteopathic Research Web, OstMed, Google.

## Results

**Figure 1:**
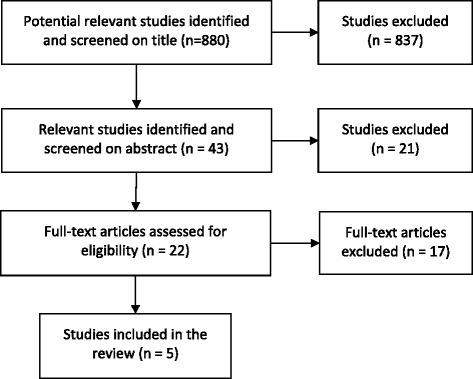
**Flow diagram for literature retrieval.**

The initial search retrieved 880 studies. After the authors had scrutinized the titles, 43 abstracts were deemed potentially relevant and were read, which in turn prompted the retrieval of 22 full-text articles which were more carefully inspected. Of these, 5 articles published between 2007 and 2013 survived the final cut and were entered into this review. The procedures involved in the screening and exclusion of studies is shown in the Flow Diagram in Figure 1. Three of these 5 studies required data transformation to allow data pooling. Since Arzola et al [1] reported intervertebral spaces rather than SP levels corresponding to the IAS, the number of participants with the IAS at this intervertebral space was divided equally among the vertebral levels above and below. Although Haneline et al [40] reported the IAS to correspond to the upper vertebral body, lower vertebral body, or intervertebral space; enough methodological detail was supplied to heuristically map the data to corresponding SPs. Although Kim et al [3] examined the participants in the “epidural position” (seated, back arched, neck flexed, arms across the chest), the authors were able to convert their findings to what would have been obtained in “anatomical position” (i.e., thorax fully upright, palms anterior) simply by subtracting one vertebral level from each reported data point, since trunk flexion has been found to raise the scapula by about one level [4]. This 1-level cephalad shift of the scapular position in flexion was also reflected in the data of Arzola et al [1], who reported data for both the epidural and anatomical positions.

Three of the studies [1,3,4] drew a line connecting the left and right IASs, defining the closest SP to this line as corresponding to the IAS. One study [43] did not specify which IAS was used and apparently used either, while another study [40] separately reported the location of both the left and right IASs relative to the spine. In tabulating the data for this latter study, the authors used an average of the left and right IAS positions, resulting in some non-integral values for the SP level. When this was the case, the number was rounded to the nearest integer for the purpose of data pooling. Since rounding protocols would on average distribute the errors in both directions, the authors are confident this loss of accuracy did not significantly alter the estimated spinal levels. Table 2 summarizes the data (transformed as necessary) from the 5 retrieved studies, and Table 3 their quality ratings [41,42]. The quality ratings for all of the included studies were 11 or higher and were considered acceptable.

**Table 2:** **Studies mapping the IAS to a spinal landmark in the upright position**

**Study**	**Sample size, participant demographics**	**Reference standard and method**	Location IAS relative to thoracic SP*	**Method of transforming data, if any**	**ModifiedQUADAS score (n/14)**
Cooperstein, 2007 [43]	n = 34, 59% male; mean Y/O = 26; all healthy	Radio-opaque marker placed on ISA, compared with spinal radiography	T6: 5.9%	n/a	11
			T7: 17.6%		
			T8: 47.1%		
			T9: 26.5%		
			T10: 2.9		
Haneline, 2008 [40]	n = 50, 50% male; mean Y/O = 47.5; health status unknown	Radiographic mensuration of scapula and spine	T6: 8.0%	Left and right scapular positions averaged	n/a
			T7: 26.0%		
			T8: 56.0		
			T9: 10.0%		
Teoh, 2009 [4]	n = 104, 54.8% male; mean Y/O; receiving chest radiography	Radio-opaque marker placed on ISA, compared with spinal radiography	T6: 1.0%	n/a	14
			T7: 9.6%		
			T8: 30.8%		
			T9: 36.5%		
			T10: 16.3%		
			T11: 5.8%		
Arzola, 2011 [1]	n = 55, 41.8% male; mean Y/O 30.7; all healthy	Ultrasonography	T6: 3.6%	Intervertebral space findings apportioned equally to segments above and below	13
			T7: 12.7%		
			T8: 29.1%		
			T9: 29.1%		
			T10: 16.4%		
			T11: 9.1%		
Kim, 2012 [3]	n = 100, 33% male; mean Y/O = 49.3; all symptomatic, variety of conditions	Epidural insertion level as seen on radiography	T5: 1%	Subtracted 1 spinal level to account for use of epidural position	14
			T6: 1%		
			T7: 25%		
			T8: 62%		
			T9: 10%		
			T10: 1%		

*Spinal levels corresponding to IAS reported following data transformation for uniform reporting and data pooling.

**Table 3:** **QUADAS ratings**

**QUADAS ITEM**	**Cooperstein, 2009** [47]	**Teoh, 2009** [4]	**Arzola, 2011** [1]	**Kim, 2012** [3]
**Abbreviations: Y = yes, N = no, U = unclear**				
1. Was the spectrum of patients representative of the patients who will receive the test in practice?	N	Y	N	Y
2. Were selection criteria clearly described?	Y	Y	Y	Y
3. Is the reference standard likely to correctly classify the target condition?	Y	Y	Y	Y
4. Is the time period between reference standard and index test short enough to be reasonably sure that the target condition did not change between the 2 tests?	Y	Y	Y	Y
5. Did the whole sample or a random selection of the sample receive verification using a reference standard of diagnosis?	Y	Y	Y	Y
6. Did patients receive the same reference standard regardless of the index text result?	Y	Y	Y	Y
7. Was the reference standard independent of the index test (ie, the index test did not form part of the reference standard)?	Y	Y	Y	Y
8. Was the execution of the index test described in sufficient detail to permit replication of the test?	Y	Y	Y	Y
9. Was the execution of the reference standard described in sufficient detail to permit its replication?	Y	Y	Y	Y
10. Were the index test results interpreted without knowledge of the results of the reference standard?	N	Y	Y	Y
11. Were the reference standard results interpreted without knowledge of the results of the index test?	N	Y	N	Y
12. Were the same clinical data available when test results were interpreted as would be available when the test is used in practice?	Y	Y	Y	Y
13. Were interpretable/intermediate test results reported?	Y	Y	Y	Y
14. Were withdrawals from the study explained?	Y	Y	Y	Y
**Total quality score**	**11**	**14**	**12**	**14**

The Haneline study, one of the 5 articles included in this review, was not amenable to QUADAS rating and thus is not included in Table 3.

**Figure 2:**
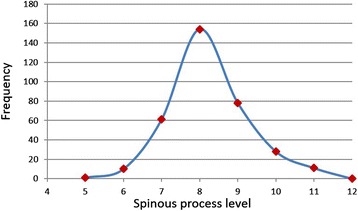
**Distribution of pooled data.**

**Figure 3:**
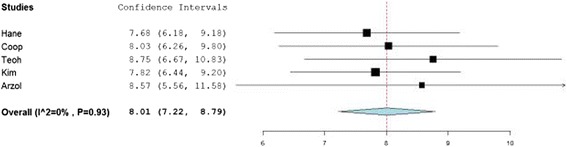
**Forest plot summarizing results of included studies.**

Following data conversions, the data from all 5 studies were combined to construct a pool of 343 participants. As seen in Figure 2, the data comprise an almost perfect bell curve centered on the T8 SP, the vertebral level whose SP most closely corresponds on average to the IAS. Normality was confirmed using the Shapiro-Wilk statistic. Two-hundred and ninety-three (85.4%) of measurements were at or within one level of the T8 SP, range T4-T11. Figure 3 is a forest plot that provides the vertebral level point estimate and 95% confidence level for each of the included studies. The areas of the tick marks denoting the point estimates are proportional to the participant size. The confidence intervals for each study overlap each other as well as the grand mean of 8.01, which almost exactly corresponds to the T8 SP. This confirms there are no statistical differences at the 95% confidence interval in the spinal level of the ISA as identified in each of the 5 studies. The very low I-squared summary statistic (I^2^ = 0%, p = 0.93) suggests very low heterogeneity among the included studies.

## Discussion

Among the 5 included articles, in all but that of Haneline et al [40] manual palpation served as an index test to identify the position of the IAS, as compared with either a radiographic or ultrasonographic reference standard. The Haneline study performed a secondary analysis of radiographs taken for reasons unrelated to that of the current study, that permitted visualization of the IAS and the SP that most closely approximated its level. Arzola [1], Teoh [4], and Kim [3] each studied the accuracy of using the IAS to locate the T7 SP, only to find that this landmark rule was not accurate. Although the failure of the benchmark rule is important to note, the purpose of our systematic review was not to assess the degree of landmark failure but rather conduct a purely anatomical secondary analysis to determine the spinal level of the IAS.

In Cooperstein et al [43], a palpator affixed a lead marker to the SP judged closest to the level of the IAS; later radiographically identified most often as the T8 SP. In Teoh et al [4] the palpator used the IAS as a landmark to locate what was expected to be the T7 SP but was later radiographically determined to correspond closest to the T8 SP. Arzola et al [1] also used the IAS to locate what was expected to be the T7 SP, using a skin marking to identify that location; ultrasononographic examination determined that location to usually lie closer to the T8 SP. Kim et al [3] inserted a catheter at spinal levels identified based on their anatomic relation to the IAS, with the patients in the flexed epidural position. After data transformation, the level of insertion most often corresponded to the T8 SP.

Accuracy in using spinal landmarks to identify segmental levels should be supported by anatomical studies. Some studies suggest that anatomical variability among patients can lead to errors in treating and charting sites of care. For example, the vertebra prominens is not always C7 [17], the L4 SP is not always at the same level as the iliac crest [18], and the IAS is not always at the T7 SP standing nor T6 SP prone [44]. The present study endeavored to both systematically review the literature on the location of the IAS in relation to the spine; and transform/pool the data from 5 individual studies to increase the precision and accuracy of their combined results. Since on average the IAS most closely identifies the T8 SP in the upright position, it is very likely that health professionals, both manual therapists and others, who have been diagnosing and treating patients based on the IAS = T7 SP rule (the conventional wisdom), have not been as segmentally accurate as they may have supposed. They either addressed non-intended levels, or made numeration errors in their charting. Arzola [1] Kim [3] and Teoh [4] reported accuracy rates of 18%, 62%, and 41% respectively in using the IAS for identifying T7. Since the IAS lies within a range spanning T4 to T11, the accuracy depends on the anatomy of the individual who is being examined. Teoh [4], comparing the accuracy of two different surface landmarks for locating the T7 SP, found the vertebra prominens (C7) to be more accurate than the tip of the scapula.

Apart from issues related to the between-individuals accuracy of the landmark rule, variability within individuals further compounds its use. The scapula on the side of the dominant arm tends to lie approximately 0.5 cm lower than the scapula on the side of the non-dominant arm [37,45], corresponding to about 0.2 vertebral levels lower [46]. Therefore the accuracy of using the IAS as a benchmark is arm-dependent.

Although a one-level error in the scapular landmark rule may not seem very clinically significant, the error may be compounded by a two-level error related to yet another inaccurate scapular landmark rule, by which the scapula supposedly rises one level in the prone compared with standing positions. Since in fact the scapula tends to move *inferiorly* about one level when the patient is prone (depending on arm position) [47], the potential exists for a three-level error when a clinician applies an intervention in the prone position based on an examination procedure performed in the upright position [24]. Clinicians who intend to treat spinal levels identified by upright examination procedures (including thermography, manual muscle testing, static/motion palpation, and x-ray) in the prone position may miss their target by 2-3 spinal level. Although we do not *assume* that the outcome of chiropractic or other types of manual care for spinal complaints is made better by identifying misalignments accurately, manual therapists and other health professionals who do believe such information improves the outcome of care are now faced with an interesting paradox. They must either consider their clinical outcomes to have been suboptimal due to targeting errors, or derive an alternative explanation for their presumed good clinical outcomes, at least in relation to spinal segments that were identified by using the IAS as a landmark.

Irrespective of whether misdiagnosing the level of a misaligned or dysfunctional segment results in a sub-optimal clinical outcome, issues may arise if or when another or the same clinician attempts to intervene on the incorrectly charted level on another day [23]. Level misidentification may also be problematic in attempting to track clinical changes over time. Accurate palpation is crucial when the practitioner is attempting to correlate physical examination findings with the results of an imaging study, in order to decide upon the clinical relevance of manual examination findings [23].

### Limitations of the study

The QUADAS instrument was used to rate the quality of 4 of the included studies, each of which compared palpatory results with a reference standard. However, it could not be used to rate Haneline [40], since this purely anatomical study did not include a palpation arm. QUADAS can only be used to assess the comparative validity of an examination method to a reference standard. Using QUADAS as we did to assess the quality of articles used in a secondary analysis is not common.

The need to transform the data in 3 of the studies may have introduced some loss of accuracy. The process of synthesizing data from studies that use different methods is error-prone; the authors did their best to make the necessary transformations with fidelity. When a study reported the location of the IAS in relation to a spinal structure other than the SP, the authors either assigned it to the nearest SP or apportioned it equally to the levels above and below, reducing the risk of systematic bias. The individual studies enrolled participants who were rather different in their demographic characteristics, thus warranting caution in drawing comparisons between studies. Conversely, the conclusions to be drawn from the pooled data are made more credible because data pooling created a rather heterogeneous participant mix, with both healthy and symptomatic, younger and older, participants.

Although the authors are aware of no data on how commonly the IAS is used to identify spinal sites in the manual therapy professions, the existence of abundant examples in the literature [43] suggests this practice is common. The existence of several studies by anesthetists determining the relation of the scapula to the spine lends to this impression. It remains to be seen whether counting up from pelvic landmarks leads to more accurate identification of thoracic spinal levels than using the scapular location as a landmark. Palpating the posterior superior iliac spines (PSISs) has been shown to be more useful than palpating the iliac crests to locate lumbar spinal levels [19], presumably because crest levels show more anatomical variation than PSIS levels [18].

## Conclusion

Manual therapists (and other health professionals) use spinal landmarks for a variety of different purposes: corroborating a specific nerve root or other segmental pathology, accurate identification of an intended site of care, accurate positioning of the clinician’s contact hand during spinal manipulation and other spinal therapies, and documentation as to the spinal level found subluxated/dysfunctional and/or treated. Although the clinical importance of attaining specificity in the identifying and treating spinal sites of care remains controversial in the manual therapy setting [48], the capacity to attain specificity is a prerequisite for accomplishing the clinical studies that would confirm or refute its importance. Based especially on Teoh’s findings [4] using the IAS may be less preferred than using the location of vertebra prominens to identify thoracic spine locations. As a non-fixed structure that may be said to “float” over the rib cage, considering the anatomical variability that follows from that, the scapula does not constitute an acceptable landmark for identifying spinal levels.

Practitioners of manual therapy, orthopedic medicine, primary medical care, neurology, anesthesiology, nursing, and acupuncture should be interested in this updated information on the anatomical relation of the scapula and spine. Anatomically incorrect landmark benchmarks will hinder the accurate identification of spinal sites of clinical interest, beyond what is to be expected due to examiner error and variation among patients. It may be of some value to systematically review the literature addressing other commonly used spinal landmark rules.
